# A PDA@ZIF-8-Incorporated PMIA TFN-FO Membrane for Seawater Desalination: Improving Water Flux and Anti-Fouling Performance

**DOI:** 10.3390/membranes14120272

**Published:** 2024-12-16

**Authors:** Yu Ma, Rui Jia, Zhen-Liang Xu, Aida Aibulatova, Xiao-Gang Jin, Yin-Xin Fang, Ming-Xiao Zhang, Sun-Jie Xu

**Affiliations:** 1 State Key Laboratory of Chemical Engineering, Membrane Science and Engineering R&D Lab, Chemical Engineering Research Center, School of Chemical Engineering, East China University of Science and Technology, 130 Meilong Road, Shanghai 200237, China; 2 Shanghai Electronic Chemicals Innovation Institute, East China University of Science and Technology, Shanghai 200237, China; 3 School of Materials Science and Engineering, East China University of Science and Technology, Shanghai 200237, China

**Keywords:** polyisophenylbenzamide, thin-film nanocomposite, forward osmosis, PDA@ZIF-8

## Abstract

Forward osmosis (FO) technology, known for its minimal energy requirements, excellent resistance to fouling, and significant commercial potential, shows enormous promise in the development of sustainable technologies, especially with regard to seawater desalination and wastewater. In this study, we improved the performance of the FO membrane in terms of its mechanical strength and hydrophilic properties. Generally, the water flux (*J_w_*) of polyisophenylbenzamide (PMIA) thin-film composite (TFC)-FO membranes is still inadequate for industrial applications. Here, hydrophilic polydopamine (PDA)@ zeolitic imidazolate frameworks-8 (ZIF-8) nanomaterials and their integration into PMIA membranes using the interfacial polymerization (IP) method were investigated. The impact of PDA@ZIF-8 on membrane performance in both pressure-retarded osmosis (PRO) and forward osmosis (FO) modes was analyzed. The durability and fouling resistance of these membranes were evaluated over the long term. When the amount of ZIF-8@PDA incorporated in the membrane reached 0.05 wt% in the aqueous phase in the IP reaction, the *J_w_* values for the PRO mode and FO mode were 12.09 LMH and 11.10 LMH, respectively. The reverse salt flux (*J_s_)/J_w_* values for both modes decreased from 0.75 and 0.80 to 0.33 and 0.35, respectively. At the same time, the PRO and FO modes’ properties were stable in a 15 h test. The incorporation of PDA@ZIF-8 facilitated the formation of water channels within the nanoparticle pores. Furthermore, the *J_s_*/*J_w_* ratio decreased significantly, and the FO membranes containing PDA@ZIF-8 exhibited high flux recovery rates and superior resistance to membrane fouling. Therefore, PDA@ZIF-8-modified FO membranes have the potential for use in industrial applications in seawater desalination.

## 1. Introduction

In the 21st century, the demand for water resources is escalating as human society progresses, underscoring these resources’ critical strategic importance [1]. Although water covers 71% of the Earth’s surface, freshwater constitutes only about 3%, and a significant portion is locked in glaciers and ice caps [2,3]. This scarcity of accessible freshwater resources has made desalination a vital method of supplementing the supply [4]. Reverse osmosis (RO) has emerged as the leading desalination technique due to its ability to effectively remove pollutants and salts from water [5]. However, the substantial energy consumption associated with seawater desalination has prompted researchers to seek alternative methods [6]. Forward osmosis (FO) is a membrane-based separation process in which osmotic pressure differences between the feed solution (FS) and draw solution (DS) are exploited [7]. FO has distinct advantages over RO, such as inducibility without additional external pressure, lower energy consumption, and reduced membrane fouling [8,9,10]. Consequently, FO has become a focal point in research aimed at enhancing seawater desalination [11,12].

Interfacial polymerization (IP) is a process commonly used for fabricating a thin-film composite (TFC) membrane’s polyamide (PA) layer. The most common way of fabricating FO membranes involves the IP method, using an m-phenylenediamine (MPD) monomer in the aqueous phase and a 1,3,5-benzoylchloride (TMC) monomer in the organic phase to develop a dense PA layer for separation [13]. However, internal concentration polarization (ICP) [14] and membrane fouling [15] pose significant challenges for TFC-FO membranes. Therefore, in recent years, some researchers have explored methods of addressing these issues. The methods of reducing ICP and membrane contamination primarily involve modifying the properties of the membrane substrate [16,17,18] and incorporating organic substances and particulate matter during the IP process [19]. The introduction of nanoparticles is the most commonly employed modification technique. Introducing UiO-66 [20], WS_2_-Cys-UiO-66-(CO_2_H)_2_ [21], and other nanoparticles into the membrane or PA layer to fabricate thin-film nanocomposite (TFN) membranes has been demonstrated to mitigate ICP effects and thus enhance the membrane’s water flux (*J_w_*). Compared to TFC-FO membranes, TFN-FO membranes suffer from fewer scaling issues and offer nearly reversible fouling processes [22], thereby mitigating ICP effects. Zeolitic imidazolate frameworks-8 (ZIF-8), a zeolitic imidazolate framework material with a porous structure, has been widely employed in TFN membranes [23]. However, there are relatively few studies on the introduction of ZIF-8 into FO membranes. Mehrzad Arjmandi et al. [24] introduced ZIF-8 into the PA layer via IP reaction using PES as the substrate. The *J_w_* was reduced compared to that for FO without ZIF-8. Furthermore, due to its instability in water [25], it is necessary to find a hydrophilic substance with which to coat the surface of ZIF-8 to enhance its water stability for application in FO seawater desalination processes. Polydopamine (PDA) exhibits excellent adhesion properties and good hydrophilicity [26], making it suitable for modifying ZIF-8 and incorporating it into membranes to enhance membrane separation performance.

The use of new high-molecular-weight polymers [27] is another approach to enhancing FO performance. Cellulose triacetate (CTA) [28] and polyethersulfone (PES) [29] are widely utilized in FO membranes due to their high hydrophilicity. Reshma Lakra et al. [30] achieved a high *J_w_* of 13.2 LMH in FO membranes prepared using CTA as the polymer. However, CTA membranes suffer from drawbacks such as low water permeability, membrane fouling, and limited biodegradability [31], sparking the search for new high-molecular-weight polymers for FO membrane preparation. Polyisophenylbenzamide (PMIA), a type of polymer, possesses a unique molecular and crystalline structure, allowing it to provide thermal resilience, chemical resistance, increased water affinity, and outstanding mechanical strength [32]. Sun et al. [33] used PMIA to fabricate FO membranes, achieving a *J_w_* of 8.62 LMH in FO mode, with superior mechanical and anti-fouling properties.

In this case, PDA@ZIF-8 was synthesized and coated with PDA. For the synthesis of PDA@ZIF-8, we used f novel means, namely, a methanol–water mixture, in which a Tris-HCl buffer solution (pH = 8.5) was used to promote dopamine self-polymerization. When ZIF-8 was added to the system, its own water stability was increased. Unlike other metal–organic frameworks (MOFs) synthesis techniques, this one only needs to be carried out at room temperature. The nanoparticles were integrated into the PA layer of the PMIA TFC-FO membrane, using the IP method to enhance performance. The effect of the PDA@ZIF-8 content on performance in both PRO and FO modes was comprehensively examined. Stability evaluations for *J_w_*, reverse salt flux (*J_s_*), and extended operational performance were carried out utilizing a bespoke cross-flow system. After we evaluated these performance parameters, the water flux recovery rate (FRR) was assessed and calculated following cleaning with deionized water. The membrane’s resistance to fouling was evaluated using humic acid (HA). The results indicated that the *J_w_* of the membrane incorporating PDA@ZIF-8 increased by 38.33% in PRO mode and 35.70% in FO mode compared to that for the membranes that had not been modified, and the *J_s_*/*J_w_* value was significantly reduced. Moreover, the membrane demonstrated strong anti-fouling capabilities. For example, the FRR was approximately 90% after cleaning. This study provides valuable insights for advancing forward osmosis membranes based on PMIA.

## 2. Experimental Section

PMIA was sourced from DuPont, Wilmington, DE, USA. Anhydrous LiCl (≥99.0%), dopamine hydrochloride (DA, ≥98.0%), n-hexane (CP, ≥96.0%), m-phenylenediamine (MPD, ≥98.0%), and Tris-HCl (≥98%) were obtained from Shanghai Maclin Biochemical Technology Co., Ltd. Shanghai, China, Polyvinylpyrrolidone (PVP, K30) and N,N-dimethylacetamide (DMAc, ≥99.0%) were supplied by Shanghai Titan Technology Co., Ltd. Shanghai, China, Methanol (≥99.5%), 1,3,5-benzoyl chloride (TMC, ≥98.0%), hydrochloric acid (HCl, 36–38%), NaCl (≥99.5%), and 2-methylimidazole (≥98%) were also acquired from Shanghai Titan Technology Co., Ltd. Polyethylene glycol (PEG, Mw = 400) was procured from Shanghai Lingfeng Chemical Reagent Co., Ltd., Shanghai, China, while Zn(NO_3_)_2_·6H_2_O was purchased from Sinopod Chemical Reagent Co., Ltd. Shanghai, China, Humic acid (HA, ≥97.0%) was obtained from Hefei BASF Biotechnology Co., Ltd. Hfei,China, and NaOH (≥98.0%) was acquired from Shanghai Aladdin Biochemical Technology Co., Ltd. Shanghai, China.

### 2.1. Preparation of PDA@ZIF-8 Nanoparticles

A total of 6.6 g 2-methylimidazole was dissolved in 100 mL of methanol and stirred magnetically for 30 min. Meanwhile, in a different container, 3 g of Zn(NO_3_)_2_·6H_2_O was mixed with 100 mL of methanol and subjected to magnetic agitation for the same duration. The two solutions were then mixed and subjected to magnetic stirring for 1 h, at which point complete precipitation took place. The mixture was subsequently centrifuged (at 8000 rpm for 10 min) to separate the solid from the liquid phase. The solid, having been rinsed three times with methanol, was subsequently subjected to thermal drying at 60 °C for 12 h. The resultant ZIF-8 nanoparticles were then gathered via milling with a mortar.

A total of 0.1 g ZIF-8 was dispersed in a solution composed of 50 mL of methanol and 0.1 mol/L of Tris-HCl (pH = 8.5), into which 0.4 g of DA was subsequently added. This mixture was then subjected to magnetic stirring for 1 h until thoroughly mixed. After the reaction took place, the solid and liquid phases were separated via centrifugation (10,000 rpm, 10 min). An oven was set to 40 °C, and the solid was put in it and dried for 12 h. After drying, the solid was ground into nanoparticles and named PDA@ZIF-8.

### 2.2. Fabrication of the PMIA Substrate

A casting solution was prepared by dissolving 6 g of PEG-400, 6 g of anhydrous LiCl, and 3 g PVP in 75 g DMAc. After complete dissolution, 10 g of dried PMIA was added incrementally in five aliquots. The resulting solution was subjected to continuous magnetic stirring in a thermostat-controlled water bath set at 80 °C for 15 h. Subsequently, the solution was left to stand at normal atmospheric temperature until all the bubbles had dissipated. The solution was subsequently moved into a syringe and deposited in an oven set to 80 °C to remove any residual bubbles until the solution was completely free of bubbles. A syringe was used to dispense a 4-milliliter aliquot of the solution onto a sterile glass panel. The membrane was uniformly applied with a modified scraper to attain a thickness of 200 μm. The glass plate supporting the membrane was then submerged in a deionized (DI) water bath at a controlled temperature, which was set to 25 °C, thereby allowing the membrane to detach automatically. Following detachment, deionized water was prepared in a container for membrane immersion, which lasted 24 h with a minimum of three water changes to effectively eliminate any remaining solvent, and the container was replenished with deionized water to preserve the membranes.

### 2.3. Fabrication of the PMIA TFC-FO and TFN-FO Membrane

**Figure 1:**
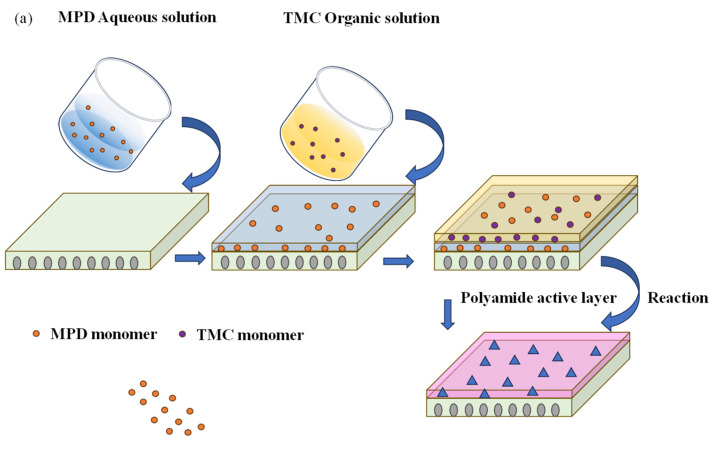

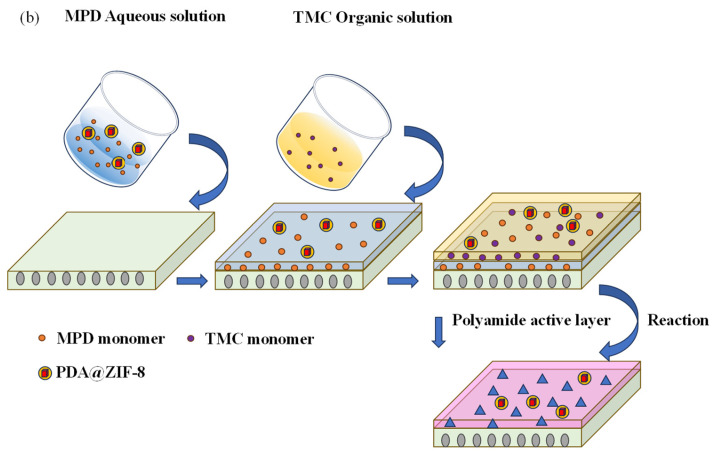
Diagram illustrating the process of membrane fabrication. (**a**) TFC-FO membrane and (**b**) TFN-FO membrane.

A 0.1 wt% solution of TMC in n-hexane was created and stored in a volumetric flask. Also, a 2.0 wt% solution of MPD in an aqueous medium was prepared. MPD solution was poured onto the membrane, and the membrane stayed submersed in it for 3 min. Subsequently, visible water droplets were removed from the membrane using an air knife, and then the organic solution was applied to the membrane and allowed to react for one minute. The specimen was subjected to thermal treatment in an oven at 60 °C for a duration of 5 min. This step aided in the removal of unreacted solvents and monomers from the membrane, leading to the formation of the TFC-FO membrane. The TFN-FO membrane underwent a preparation process similar to that used for the TFC-FO membrane. However, compared to the membrane without adding nanoparticles, the difference was that the mass fractions of PDA@ZIF-8 were incorporated into the MPD aqueous solution (from 0.01 wt% to 0.07 wt%, increment by 0.01). In the subsequent steps of the preparation process, as illustrated in Figure 1a,b, we followed the same procedures used for the TFC-FO membrane.

### 2.4. Characterization of Membranes

#### 2.4.1. Characterizations of PDA@ZIF-8 Nanoparticles

Attenuated total reflection Fourier transform infrared spectroscopy (ATR-FTIR, Thermo Fisher Scientific Nicolet iS20, Waltham, MA, USA) was employed to analyze the functional groups of ZIF-8 and PDA@ZIF-8. The scanning wavelength range was 400–4000 cm^−1^. Scanning electron microscopy (SEM, FEI Nova Nano, Stanford, CA, USA) and X-ray diffraction (XRD, Rigaku SmartLab SE, Tokyo, Japan) were utilized to observe the microstructural differences between the two MOFs in order to investigate their crystalline structures.

#### 2.4.2. Characterizations of the PMIA FO Membrane

ATR-FTIR and X-ray photoelectron spectroscopy (XPS, Thermo Fisher Nexsa, Waltham, MA, USA) were employed to analyze the surface chemical structure and elemental composition of the membrane. To further investigate the membrane’s microstructure, SEM was used to observe its surface and cross-sectional morphology, while atomic force microscopy (AFM, Bruker Dimension Icon, Karlsruhe, Germany) was utilized for an additional analysis of surface roughness. A contact angle goniometer (JC2000D) was used to measure the water contact angle of the membrane, assessing its hydrophilicity. A zeta potential analyzer was employed to test the membrane surface’s electrical properties.

### 2.5. Evaluation of FO Performance

The *J_w_* and *J_s_* of both the TFC-FO and TFN-FO membranes were assessed using a custom-built cross-flow filtration device. This device features an effective test area of 24.18 cm^2^ and includes two symmetrical flow channels with a depth of 1 cm each. In these experiments, a 1M NaCl solution served as a DS, while deionized water was used as an FS. Membrane performance was assessed in PRO mode, with the PA layer facing the DS, and in FO mode, with the PA layer facing the FS.

The water flux of the membrane is calculated using Formula (1) (*J_w_* LMH):

(1) $$J_{w}=\frac{∆m}{\rho ∆tA}$$

where Δ*m* is the reduction of feed mass (g), *ρ* is the density of deionized water (g/L); *A* is the effective test area of FO membrane (m^2^); and Δ*t* is the test time (h).

The reverse salt flux of the membrane was calculated using Formula (2) (*J_s_* LMH):

(2) $$J_{s}=\frac{C_{t}V_{t}-C_{0}V_{0}}{A∆t}$$

where *C*_0_ is the initial concentration of DS (g/L); *C_t_* is the concentration of DS at time *t* (g/L); *V*_0_ is the initial volume of FS (L); the volume of FS at time *V_t_* (L); *A* is the effective test area of FO membrane (cm^2^); and Δ*t* is the test time (h). The concentration of the solute was tested by measuring the electrical conductivity.

FO membrane performance was evaluated in both PRO mode and FO mode continuously for 15 h, with data collected at 30 min intervals. Following each experiment, DI water was used to rinse membranes for 5 min, and the FRR of the membranes was subsequently re-evaluated.

The fouling properties of the FO membranes were assessed. To simulate a polluted environment, 10 mg of humic acid (HA) was added to a 1 M NaCl solution. The HA was dissolved in 0.1 M NaOH and adjusted to pH 8.0 with 0.1 M HCl. It was noted that HA can affect flux in membrane separation [33]. Performance in both PRO mode and FO mode was evaluated over 6 h, with data recorded every 30 min. After the experiment, the membranes were flushed with DI water for 5 min, and the FRR of the membranes was reassessed.

At the conclusion of the long-term stability or fouling experiments, the water flux recovery rate was used to assess how well the flux recovered after the cleaning step. It can be calculated using Formula (3) (*FRR*, %):

(3) $$FRR=\frac{J_{r}}{J_{0}}\times 100\%$$

where *J_r_* represents the flux after cleaning at the end of the long-term membrane stability or membrane fouling experiment (LMH), and *J*_0_ denotes the initial flux (LMH).

## 3. Results and Discussion

### 3.1. Characterization of ZIF-8 and PDA@ZIF-8 Nanoparticles

**Figure 2:**
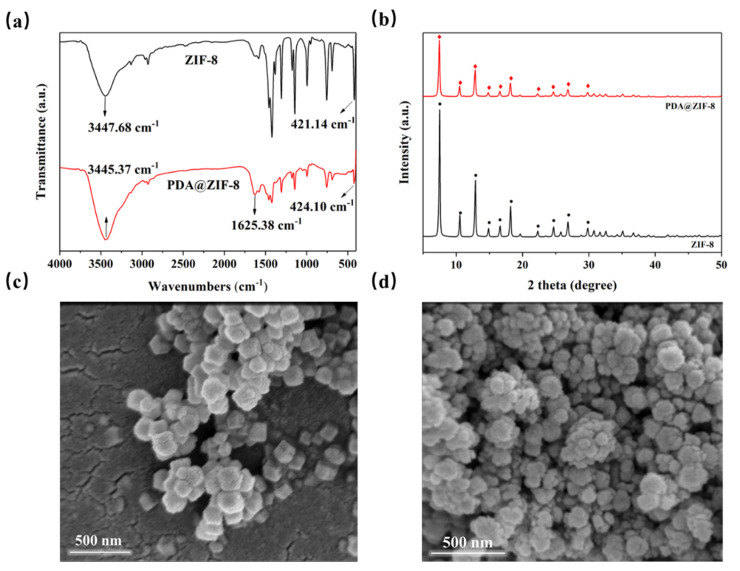
Characterization of ZIF-8 and PDA@ZIF-8. (**a**) ATR-FTIR; (**b**) XRD; (**c**) SEM image of ZIF-8; and (**d**) SEM image of PDA@ZIF-8.

Functional group analysis of ZIF-8 and PDA@ZIF-8 was conducted using ATR-FITR, and the results are illustrated in Figure 2a. The peaks detected at 421 cm^−1^ and 424 cm^−1^ originate from Zn-N stretching vibrations [34], while the peaks at 3447 cm^−1^ and 3445 cm^−1^ stem from N-H stretching [35], indicating successful synthesis of ZIF-8. The peak at 1625 cm^−1^ in PDA@ZIF-8 can be attributed to the C=C resonance of the PDA benzene ring [36]. These functional group analysis results confirm that ZIF-8 was successfully coated with PDA. The XRD results (Figure 2b) indicate that ZIF-8 exhibited distinct characteristic diffraction peaks at 2θ of 7.4°, 10.4°, 12.7°, 14.7°, 16.4°, 18.0°, 22.1°, 24.5°, 26.7°, and 29.6°, which correspond to the (011), (002), (112), (022), (013), (222), (114), (233), (134), and (044) planes, respectively [37]. This finding proves that ZIF-8 features a sodalite-type crystal structure and maintains high crystallinity [38]. The XRD patterns of PDA@ZIF-8 closely resemble those of ZIF-8, suggesting that the crystal structure of ZIF-8 remained mostly intact after PDA coating [39]. SEM (Figure 2c,d) characterization further confirmed that the two nanoparticles exhibit well-defined crystalline structures.

### 3.2. The Chemical Composition of the FO Membrane

ATR-FTIR and XPS were used to analyze functional groups and chemical elements that exist on the surfaces of the membranes.

**Figure 3:**
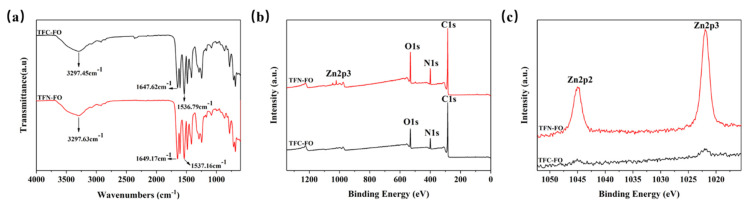
ATR-FTIR and XPS spectra of the TFC-FO and TFN-FO membranes. (**a**) ATR-FTIR; (**b**) XPS full spectrum; and (**c**) XPS zinc elemental spectrum.

Figure 3a displays the ATR-FTIR spectra of the TFC-FO and TFN-FO membranes in the 400 to 4000 cm^−1^ range. Both membranes exhibit prominent peaks near 3297 cm^−1^ and 1537 cm^−1^, which are characteristic peaks of PMIA [40]. A peak near 1649 cm^−1^ was observed for the TFC-FO and TFN-FO membranes; it can be attributed to C=O stretching in the PMIA sublayer and the PA layer [41,42,43]. These findings indicate a PA layer was successfully developed on the PMIA substrate through the IP method. The absorption spectra of the TFC-FO and TFN-FO membranes exhibit similarity, potentially due to overlap between the C=C peak (1607 cm^−1^) and the NH peak [44,45,46], as well as the low content of PDA@ZIF-8 on the membrane surfaces, preventing clear detection of characteristic peaks [40]. To further characterize the introduction of PDA@ZIF-8 into the membranes, XPS was used to conduct the elemental analysis of the two types of membranes. The full spectra are illustrated in Figure 3b, and the membranes’ elemental content is listed in Table 1. Compared to the TFC-FO membrane, the nitrogen content in the TFN-FO membrane rose from 8.95% to 9.67%, attributed to the introduction of nitrogen from PDA@ZIF-8 [47]. The zinc content was determined to be 0.51%. XPS fine spectrum analysis of zinc elements in both membranes is presented in Figure 3c. The TFN-FO membrane exhibited peaks at 1021.98 eV (Zn2p3) and 1044.98 eV (Zn2p2), which are slightly shifted compared to the standard ZIF-8 peaks at 1021.6 eV and 1044.7 eV [48], possibly due to electron transfer induced by PDA coating [49]. In contrast, the TFC membrane without PDA@ZIF-8 showed rough peaks in the range of 1020–1045 eV without distinct Zn2p3 and Zn2p2 peaks, indicating the absence of zinc in the TFC membrane. PDA@ZIF-8 was successfully incorporated into the membranes, as supported by these characterization results.

### 3.3. Surface Morphology and Performance Characteristics of the FO Membranes

**Figure 4:**
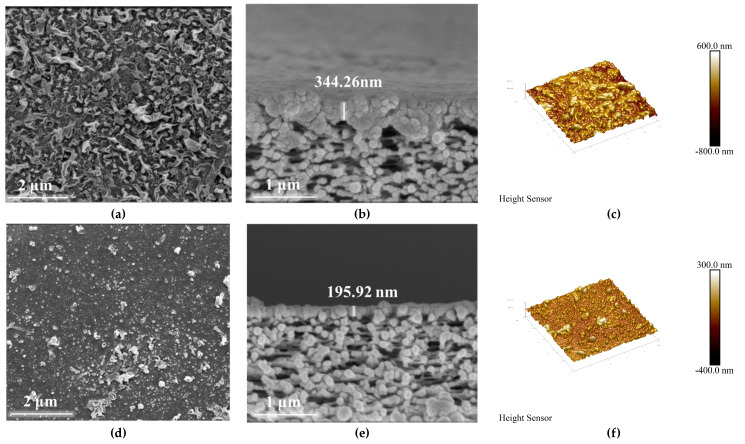
(**a**–**c**) SEM and AFM images of the TFC-FO membrane. (**d**–**f**) SEM and AFM images of the TFN-FO membrane.

Figure 4a,b illustrate the surface morphologies of the membranes. The TFC-FO membrane exhibits a leaf-like structure, which is one of the PA layer’s typical characteristics [50]. Constituting an n compatibility between the two membranes, the TFN-FO membrane shows less-distinct leaf-like features and distributed PDA@ZIF-8 crystals, with some regions displaying blocky formations of the membrane surface’s nanoparticles, likely on account of particle aggregation [51]. Cross-sectional images of the TFC-FO and TFN-FO membranes are shown in Figure 4c,d, with PA layer thicknesses of 204 nm and 344 nm. The characterization results further confirm PDA@ZIF-8’s incorporation into the membrane’s PA layer.

**Figure 5:**
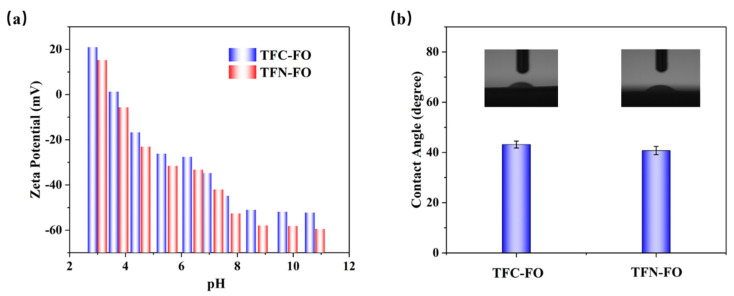
(**a**) Zeta potential measurements of the two membranes. (**b**) The DI water contact angle of the TFC-FO and TFN-FO membrane.

The hydrophilicity and surface roughness of membranes significantly impact their performance and fouling resistance in FO applications [52]. The water contact angle of a membrane is a key parameter for assessing its hydrophilicity; a larger contact angle indicates greater hydrophobicity of the membrane [53]. Figure 5b demonstrates that the two membranes’ average water contact angles are 43.2° and 40.8°. This is because of the hydrophilic properties of PDA@ZIF-8 [50], in which the membrane surface’s hydrophilicity is elevated. The results of zeta potential testing (Figure 5a) indicate that at the same pH value, the TFN-FO membrane is more negatively charged than the TFC-FO membrane, which is attributed to the introduction of carboxyl groups that have negative charges during the IP reaction incorporating PDA@ZIF-8 [54], thereby improving the membrane’s fouling resistance and separation performance [50,55]. To gain a deeper understanding of membranes’ surface morphology and roughness, AFM is often applied [51]. The experimental results are revealed in Figure 4e,f and Table 2. The membrane surface without PDA@ZIF-8 exhibited an average roughness (R_a_) of 89.3 nm, whereas the membrane incorporating PDA@ZIF-8 showed an R_a_ of 29.8 nm, suggesting that incorporating PDA@ZIF-8 enhanced the membrane’s surface morphology. A lower R_a_ value suggests potentially improved resistance to the fouling of the membrane [56].

### 3.4. The PRO and FO Properties of the FO Membrane

#### 3.4.1. Influence of the Amount of PDA@ZIF-8 Incorporated on the PRO Mode and FO Mode

**Figure 6:**
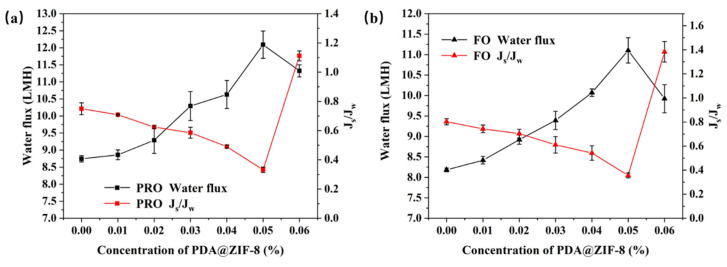
The impact of PDA@ZIF-8 loading on the functionality of the (**a**) PRO mode and (**b**) FO mode membranes.

The aqueous phase of the substrate in the IP reaction was combined with varying concentrations of PDA@ZIF-8 (ranging from 0.01 wt% to 0.07 wt%, incremented by 0.01). In this study, we sought to evaluate the impact of varying degrees of PDA@ZIF-8 inclusion on the efficacy of PMIA-FO membranes in both PRO and FO modes. The results are illustrated in Figure 6. The experimental data indicate a gradual increase in *J_w_* in the two modes as the incorporation rate of PDA@ZIF-8 rose from 0.01 wt% to 0.06 wt%. The maximum values observed are 12.09 LMH in PRO mode and 11.10 LMH in FO mode, achieved when the incorporation rate of nanoparticles was 0.05 wt%. In comparison to the membrane without PDA@ZIF-8, the *J_w_* increased by 38.33% in PRO mode and 35.70% in FO mode. The O/N ratio of the membrane decreased from 0.70 to 0.63 after the addition of PDA@ZIF-8, indicating an increase in the degree of crosslinking of the PA layer. In addition to the fact that the introduction of PDA@ZIF-8 changed the O/N ratio, this result may also be due to the interaction between the incorporation of PDA@ZIF-8 and the PA layer, which improved the degree of crosslinking of the PA layer and enhanced the separation ability of the membrane, thereby reducing the *J_s_*/*J_w_*. The SEM cross-section results showed that the thickness of the PA layer of the film decreased from 344.26 nm to 195.92 nm after the incorporation of PDA@ZIF-8. The thickness of the PA layer reduces the mass transfer resistance of the membrane. A reduction in the thickness of the PA layer contributes to the formation of micropores at the junction of the bottom membrane and the PA layer, and these micropores act as water channels during filtration. The AFM characterization results showed that the R_a_ of the membrane decreased from 89.3 nm to 29 nm. After the incorporation of PDA@ZIF-8, the local surface roughness of the membrane was larger than that of the surrounding membrane, but it was generally lower than that of the membrane without PDA@ZIF-8, further proving that the incorporation of PDA@ZIF-8 reduces the mass transfer resistance of the membrane, thereby increasing the *J_w_*. Incorporating PDA@ZIF-8 enhances the membrane’s effective surface area, promoting the diffusion of water molecules through the PA active layer [57,58,59]. The PDA coated with ZIF-8 exhibited strong hydrophilicity, thereby further enhancing the membrane surface’s hydrophilicity [60]. Nevertheless, when PDA@ZIF-8 was integrated into the aqueous phase at a concentration of 0.06 wt%, *J_w_* diminished in both modes. A large amount of particulate matter was accumulated in an area on the membrane’s surface, leading to increased resistance that neutralized the aforementioned factors, contributing to an increased *J_w_* [61]. A large amount of PDA@ZIF-8 clumps together when it is added to a water-based solution (0.07% wt%), damaging the structure of the PA layer and PMIA substrate. This leads to membrane damage and renders it unusable for applications. As the integration amount of PDA@ZIF-8 rose from 0.01 wt% to 0.05 wt%, the *J_s_*/*J_w_* ratio of both modes progressively declined. This trend can be attributed to the preferential introduction of PDA@ZIF-8 into defects in the PA layer. This addition improves the structural density of the TFN-FO membrane, thereby reducing *J_s_*/*J_w_* [62]. However, when the PDA@ZIF-8 content surpasses 0.05 wt%, significant aggregation of PDA@ZIF-8 on the membrane surface occurs. This aggregation disrupts the structure of the PA layer and substrate, leading to a significant increase in the *J_s_*/*J_w_* ratio. For this reason, the performance of the two modes deteriorates.

#### 3.4.2. The Long-Term Stability of FO Membranes

**Figure 7:**
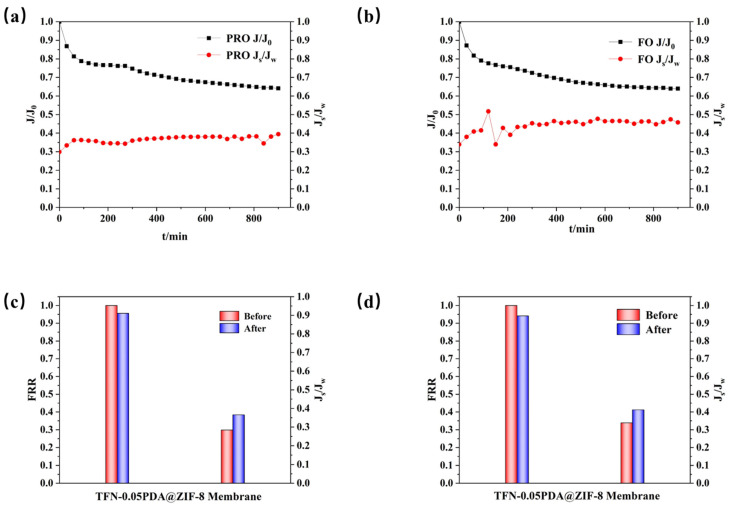
Results of the long-term stability experiment of membranes: (**a**) PRO mode and (**b**) FO mode water flux and *J_s_*/*J_w_* curves. Performance recovery bar chart of (**c**) PRO mode and (**d**) FO mode after cleaning.

The long-term stability of the FO membrane, which had the highest performance, in the PRO mode and FO mode was investigated. The results, shown in Figure 7, indicate a significant initial decrease in *J_w_* for both modes within the first 200 min. Subsequently, the rate of decrease slowed down, and stabilization was observed after 720 min. This phenomenon occurs because an ICP layer forms on the membrane’s surface, which initially reduces *J_w_*. After completing the long-term stability experiment, DI water, a commonly used solvent, was used to rinse the membrane, and performance in the two modes was re-evaluated, as illustrated in Figure 7c,d. The FRRs were 95.60% for PRO mode and 94.19% for FO mode, indicating that the membrane exhibits stable performance and can be used effectively over an extended period [63].

#### 3.4.3. Membrane-Fouling Properties

**Figure 8:**
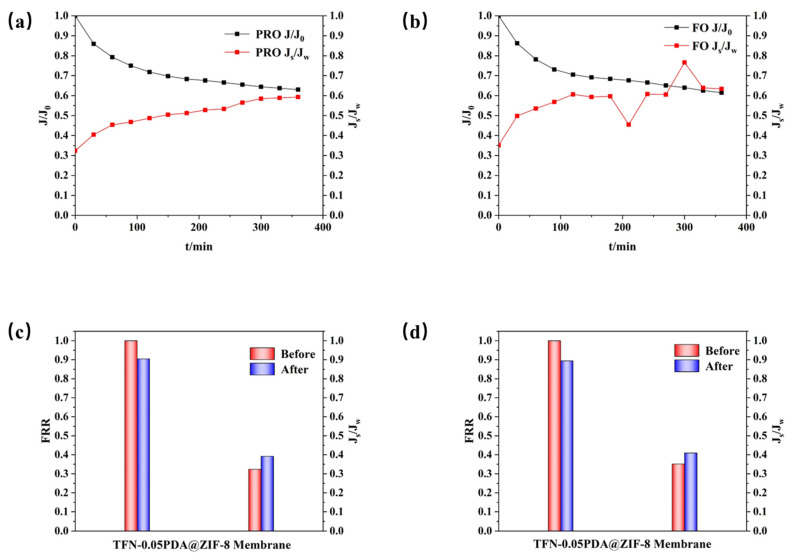
Results of the membrane fouling experiment of membranes: (**a**) PRO mode and (**b**) FO mode water flux and *J_s_*/*J_w_* curve. Performance recovery bar chart of (**c**) PRO mode and (**d**) FO mode after cleaning.

HA was selected as a model pollutant to represent organic contaminants in seawater for membrane-fouling research due to its widespread use [33]. The membrane’s resistance to pollution was studied using HA, and the experimental findings are depicted in Figure 8. In both PRO and FO modes, *J_w_* decreased by approximately 40% within 360 min when exposed to HA as a simulated pollutant. This result indicates that the presence of pollutants accelerates the ICP effect, resulting in a much faster decline in *J_w_* compared to that in unpolluted conditions. Furthermore, *J_s_* increased more rapidly when exposed to pollutants. After cleaning the membrane with DI water, the performance in the two modes was reassessed, as shown in Figure 8. It was observed that the FRRs were approximately 90% for both modes, suggesting that membrane fouling induced by HA has some degree of irreversibility [64]. However, the membrane incorporating PDA@ZIF-8 demonstrated anti-fouling properties and achieved high flux recovery after simple cleaning. These characteristics underscore its potential for use in industrial applications.

### 3.5. Comparison of FO Membrane Performance in the Literature

A comparison of the performance of FO membranes based on various substrates, commercial membranes, and this work’s membranes is summarized in Table 3. The PMIA substrate membrane with nanoparticles has a moderate *J_w_* and a lower *J_s_*/*J_w_* ratio. This *J_w_* is about twice as high as that for a commercial membrane. Thus, this study provides important insights and supports future research into and commercialization of FO membranes that use PMIA substrates.

## 4. Conclusions

In this study, PDA@ZIF-8 nanoparticles were synthesized and then incorporated into a membrane via the IP method. A TFN-FO membrane was prepared using PMIA as the substrate. The results show that the TFN-FO membrane outperformed the TFC-FO membrane that did not contain nanoparticles. The membrane modified with PDA@ZIF-8 exhibited enhanced hydrophilicity and a larger effective surface area. The research examined how different amounts of PDA@ZIF-8 affect the properties of both PRO mode and FO mode membranes. When the amount of PDA@ZIF-8 in the aqueous phase reached 0.05 wt% during the IP reaction, the *J_w_* in both PRO mode and FO mode increased by 38.33% and 35.70% compared to that for the membrane without PDA@ZIF-8. Additionally, the *J_s_*/*J_w_* value decreased noticeably, indicating improved salt rejection efficiency relative to water flux. This optimal amount of PDA@ZIF-8 was identified based on these performance enhancements. Subsequently, the long-term stability and anti-fouling capabilities of PDA@ZIF-8 membranes with the optimal dosage were investigated. The membranes exhibited stability over extended periods, and after long-term experimental cleaning, the *J_w_* recovery rate was high. Exposure to HA as a pollutant restored the *J_w_* in both PRO mode and FO mode to approximately 90%, indicating effective recovery. Moreover, under these conditions, the increase in the *J_s_*/*J_w_* value was minimal, demonstrating the membrane’s robust anti-fouling properties. This study provides theoretical insights into the development of TFN-FO membranes with PMIA as a substrate for seawater desalination. Additionally, this membrane demonstrates effective anti-fouling properties, indicating promising prospects for commercial applications.

## Figures and Tables

**Table 1:** Element concentrations of the two membranes.

Element Concentration (%)					
Membrane	C	N	O	Zn	O/N Ratio
TFC-FO	78.25	12.8	8.95	0	0.7
TFN-FO	74.43	15.34	9.67	0.56	0.63

**Table 2:** The surface roughness of the two membranes.

Membrane	R_q_ (nm)	R_a_ (nm)	R_max_ (nm)
TFC-FO	110	89.3	663
TFN-FO	38	29	302

**Table 3:** Comparison of the performance (FO mode) of different FO membranes.

Substrate	Modification	Temperature (°C)	pH	Draw Solution	*J_w_* (LMH)	*J_s_*/*J_w_*	Practical Application
CTA [28]	/	25	7.0	2 M NaCl (0.5 h)	12.8	0.53	Simulated seawater
CTA [29]	CS-AC	25	7.0	2 M NaCl (0.5 h)	19.4	0.665	Simulated seawater
PVDF [65]	/	25	7.0	1 M NaCl (0.5 h)	10.1	1.12	Simulated seawater
PSf [66]	/	25	7.0	1 M NaCl (0.5 h)	8	0.75	Simulated seawater
PSf [66]	2.0 wt%LDH/GO	25	7.0	1 M NaCl (0.5 h)	13.5	5.5	Simulated seawater
PES [67]	/	25	7.0	0.5 M NaCl (0.5 h)	9.5	0.39	Simulated seawater
PES [67]	0.5 wt%MWNTs-COOH	25	7.0	0.5 M NaCl (0.5 h)	21.8	0.57	Simulated seawater
Commercial [33]	/	25	7.0	1 M NaCl (0.5 h)	6.71	0.01	Simulated seawater
PMIA (This work)	/	25	7.0	1 M NaCl (0.5 h)	8.18	0.8	Simulated seawater
PMIA (This work)	0.05PDA@ZIF-8	25	7.0	1 M NaCl (0.5 h)	11.1	0.35	simulated seawater

## Data Availability

The raw data supporting the conclusions of this article will be made available by the authors on request.
